# Involvement of Intestinal Microbiota in Adult Neurogenesis and the Expression of Brain-Derived Neurotrophic Factor

**DOI:** 10.3390/ijms232415934

**Published:** 2022-12-14

**Authors:** Nishtha Agnihotri, M. Hasan Mohajeri

**Affiliations:** Institute of Anatomy, University of Zurich, Winterthurerstrasse 190, 8057 Zürich, Switzerland

**Keywords:** gut-brain-axis, BDNF, bacteria, neuron, neurotrophin, hippocampus

## Abstract

Growing evidence suggests a possible involvement of the intestinal microbiota in generating new neurons, but a detailed breakdown of the microbiota composition is lacking. In this report, we systematically reviewed preclinical rodent reports addressing the connection between the composition of the intestinal microbiota and neurogenesis and neurogenesis-affecting neurotrophins in the hippocampus. Various changes in bacterial composition from low taxonomic resolution at the phylum level to high taxonomic resolution at the species level were identified. As for neurogenesis, studies predominantly used doublecortin (DCX) as a marker of newly formed neurons or bromodeoxyuridine (BrdU) as a marker of proliferation. Brain-derived neurotrophic factor (BDNF) was the only neurotrophin found researched in relation to the intestinal microbiota. Phylum Actinobacteria, genus *Bifidobacterium* and genus *Lactobacillus* found the strongest positive. In contrast, phylum Firmicutes, phylum Bacteroidetes, and family Enterobacteriaceae, as well as germ-free status, showed the strongest negative correlation towards neurogenesis or BDNF mRNA expression. Age, short-chain fatty acids (SCFA), obesity, and chronic stress were recurring topics in all studies identified. Overall, these findings add to the existing evidence of a connection between microbiota and processes in the brain. To better understand this interaction, further investigation based on analyses of higher taxonomic resolution and clinical studies would be a gain to the matter.

## 1. Introduction

New neurons are formed from neural stem cells through the process of neurogenesis [1]. During the time of early development, neurogenesis reaches its maximum and subsides in most regions of the brain in the time following [2]. For a long time, researchers thought that neurogenesis stops early in life. Still, after nearly six decades of research, it is now recognized that the birth of new neurons continues throughout life at a slower pace, called adult neurogenesis [3]. Till now, adult neurogenesis has been mainly found in two areas in the brain, namely the subventricular zone of the lateral ventricles for the olfactory bulb and the subgranular zone in the dentate gyrus of the hippocampus [4]. Altman and Das were the first to discover adult neurogenesis in the hippocampus in 1965 [1]. The interest in this field has only grown in the past two decades as the introduction of staining methods using Neuronal Nuclei (NeuN) [5], Bromodeoxyuridine (BrdU) [6], and Doublecortin (DCX) [7] was instrumental in detecting newly born neurons in the brain. While BrdU staining can be used to study cell proliferation in any living tissue [8], DCX antibodies bind to microtubule-associated proteins expressed in immature neurons. Therefore, a higher specificity for neurogenesis is given [9]. To detect the general abundance and survival of mature neurons, NeuN is used. Normally, it is expressed 2–3 weeks after mitosis in most neuronal cell types and supersedes the expression of DCX [5]. Since the scope of this paper is to thematize neurogenesis, the focus was laid on DCX and BrdU expression in the hippocampus.

The regulation of adult neurogenesis is a complex interplay of both intrinsic and extrinsic factors, which range from the stem cell niche microenvironment, sex hormones, and neurotrophins to physical activity, learning, and stress [10]. In the past ten years, substantial research has been performed on neurotrophins and their importance in adult neurogenesis, as well as their influence on diseases such as Hyperalgesia, Alzheimer’s disease, and major depressive and bipolar disorder [11]. Of all neurotrophins, brain-derived neurotrophic factor (BDNF) has been most intensively studied in relation to adult neurogenesis, and multiple studies have shown that BDNF is required for neurogenesis in the subventricular zone of the hippocampus [12,13,14]. Apart from modulating neurogenesis, BDNF plays various roles in brain physiology and also has a crucial role outside of neuronal tissue such as in muscle tissue and vascular endothelial cells. In the central nervous system, this neurotrophin is involved in long-term potentiation and synaptic plasticity, where it shapes the morphology of mature neurons by promoting axonal outgrowth, dendritic arborization, and pruning. Further, deficits in BDNF signaling may contribute to the evolvement of major diseases like Alzheimer’s disease and major depressive disorder [15].

The gut microbiota has evolved to be an important contributor to neurogenesis and the production of neurotrophins. The human gut microbiota is composed of an estimated 3.8 × 10^13^ (38 trillion) microbial cells, which are segregated into more than 1000 species [16,17]. The research on microbiota is still in its infancy, and the majority of the evidence is based on preclinical studies. Interestingly, the number of bacterial species in mice microbiomes is also estimated to be around 1000. Although the number of species is similar in humans and mice, only 2.58% of the taxonomic diversity is shared between the two [18]. Nevertheless, previous studies have shown that a high percentage of gut microbial functions are shared between humans and mice, supporting the use of mouse models in human microbiota research [18,19].

Several pathways of communication between the gut microbiota and the brain have been described [20]. The most important being neural via the vagus nerve and the enteric nervous system, through cytokines on the basis of the highest density of immune cells in the gastrointestinal tract and endocrine via modulation of the hypothalamic-pituitary-adrenal axis (HPA axis) and subsequently changing glucocorticoid homeostasis [20]. Additionally, selected intestinal bacteria are capable of producing metabolites from otherwise indigestible food, including short-chain fatty acids (SCFAs) [21] and various neurotransmitters (catecholamines [22], gamma-aminobutyric acid (GABA) [23] and serotonin [24]). The vagus nerve extensively innervates gastrointestinal, respiratory, and cardiovascular organs, which is how the brain receives and provides vital information from and to these systems [25]. In vagotomized mice, DCX+ and BrdU+ cells, as well as BDNF mRNA levels, were decreased in the adult hippocampus [26,27], showing the relevance of this pathway of communication. It is long known that stress, especially chronic stress, is associated with numerous disorders like major depressive disorder and anxiety but can also have a negative impact on the developing brain [28]. A key player in stress modulation is the HPA axis, which on the other hand, can be interfered with by the gut microbiota [20]. Stress stimulates the HPA axis, leads to a release of adrenocorticotrophin in the pituitary gland, and further results in a higher secretion of corticosterone [29]. No study so far examined the effect of the gut microbiota on the HPA axis and hippocampal neurogenesis or BDNF, but changes in N-methyl-D-aspartic acid (NMDA) and serotonin_1A_ receptors have been documented in germ-free mice. Both receptors are known to influence the HPA axis and are modulators of neuronal plasticity in the hippocampus [30]. SCFAs are the most examined bacterial metabolites and consist mainly of acetate, propionate, and butyrate [31]. They act upon various gut-brain pathways, including the vagal, immune, and endocrine [32]. In an in vitro study, increased growth rate and elevated expression of proliferation-related genes were demonstrated in human neural progenitor cells following their exposition to physiologically relevant concentrations of SCFAs. This data suggests a direct effect of these metabolites on neuronal growth [33]. A recent review on the mechanisms of action of bacterial metabolites in the pathogenesis of seven common brain diseases (attention deficit hyperactivity disorder, autism spectrum disorder, schizophrenia, Alzheimer’s disease, Parkinson’s disease, major depressive disorder, and bipolar disorder) found SCFAs to be reduced in most of the diseases discussed. Additionally, a shift of the microbiota composition towards a pro-inflammatory phenotype was consistently observed at a higher taxonomic rank [34].

In this review, we aimed to systematically analyze the connection between murine intestinal microbiota and neurogenesis as well as BDNF mRNA or protein levels in the hippocampus. The focus was laid on intestinal microbiota composition. We report here several positive and negative modulators of neurogenesis and BDNF expression on phylum, genus, and family levels.

## 2. Materials and Methods

**Figure 1:**
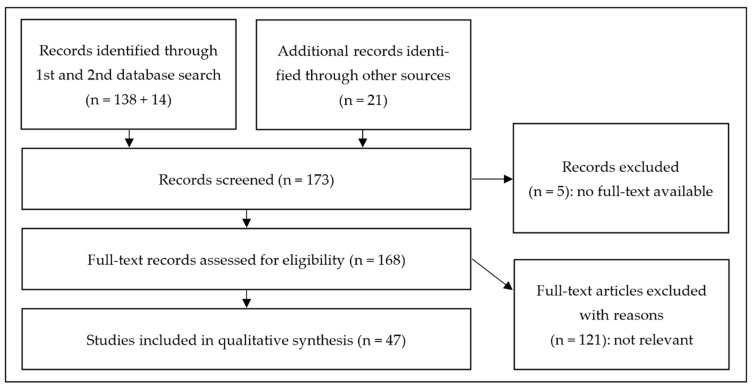
Methodical approach of our review according to PRISMA criteria.

This systemic review was conducted according to the Preferred Reporting Items for Systematic Reviews and Meta-analysis (PRISMA) guidelines (Figure 1) [35]. The main objective was to understand and summarize the available data on the influences of gut microbiota composition on neurogenesis and neurotrophic factors within the central nervous system, especially neurogenesis in the hippocampus.

PubMed and SCOPUS searches were conducted until 1 December 2021 with the following MeSH and search terms: “gut microbiota/microbiome/bacteria” and “intestinal microbiota/microbiome/bacteria” combined with “neurogenesis”, “neurotrophic factors”, and “central nervous system”, delivering 138 hits after removing duplicates. Twenty-one additional records with relevant information were individually selected from the list of references of the initially identified papers or identified by other sources. To update the reviewed literature, a second search was conducted on 1 December 2022, delivering 14 hits, of which no additional studies could be selected for this review.

We focused on bacterial taxa and therefore excluded data on viruses, archaea, and fungi, as well as microbiomes other than the intestinal microbiota.

The inclusion criteria were the following:

- Analysis of the bacterial taxa by sequencing methods
- Comparison of the gut microbiota to controls
- Measurement of neurogenesis in the hippocampus through immunohistology
- Measurement of neurotrophic factors mRNA or protein through immunohistology or in situ hybridization
- paper written in English, published in a peer-reviewed journal, and available as a full-text PDF

By including only preclinical rodent studies, we ruled out the potential confounding influence of different host species. However, to provide a broad overview of the published data on the topic, the effort was made to include all studies using varying methods for detecting neurogenesis or neurotrophic factors and sequencing the intestinal microbiome, which led to a heterogeneous group of reports.

Most papers were dated from 2010 to 2021. Five papers with no full-text availability were excluded. Further, 107 articles were excluded based on the lack of relevance to the topic. In total, 47 studies were included in the qualitative synthesis.

## 3. Results

### 3.1. Germ-Free Mice Studies

In total, fifteen publications were found addressing the role of microbiota on neurogenesis and neurotrophin expression in germ-free animals. The results are presented as the actual increase or decrease of DCX+ or BrdU+ cells or BDNF levels in the hippocampus. Results demonstrating no significant changes are mentioned as “Unchanged” in the two tables following.

The majority of studies in germ-free mice found a decrease or no change in neurogenesis [36,37,38], while only two studies showed increased neurogenesis [36,39] (Table 1). Ogbonnaya et al. [39] discovered increased BrdU+ cells in germ-free mice at ten weeks of age, specifically in the dorsal hippocampus. Conventionalization through cohabitating could not reduce the number of BrdU+ cells, which suggests that there is a sensitive period during which the microbiota interacts with the developing brain. In fact, the postnatal and adolescent period appears to be a critical window for microbiota modification to ensure optimized brain development and good mental health [40]. The second study [36] evaluated the effects of age and sex in germ-free mice on neurogenesis and found that female mice at the age of 8 weeks had increased DCX+ and BrdU+ cells in the hippocampus compared to conventional female mice, replicating the results of Ogbonnaya et al. To compare the change rate across males and females, the same study examined the percentual change in neurogenesis with the 4-week-old mouse as the baseline. For both sexes, they found increased change rates which gave the appearance that neurogenesis at 8 weeks compared to 4 weeks was increased in males and females. In female mice, the increase between the two time points was significant, while in germ-free male mice, opposite to conventional male mice, no significant increase was observed between 4 to 8 weeks of age [36]. In conclusion, these data suggest that the natural decline in DCX+ and BrdU+ cells, which occurs between weeks 4 to 8 in conventionally raised mice, was delayed in both male and female germ-free mice. Since a previous study found elevated corticosterone in germ-free mice [41] and the administration of corticosterone to nursing rodents led to a similar delayed decline of neurogenesis in the offspring [42], Scott et al. discussed differences in serum corticosterone concentrations in germ-free mice as a potential explanation for the present result in altered neurogenesis.

Scott et al. [36] also discovered an early decrease in DCX+ and BrdU+ cells at 4 weeks in germ-free male mice compared to controls. The sex- and age-dependent differences in neurogenesis between germ-free and controls normalized as animals aged to 12 weeks. This points towards the assumption that the effect of absent microbiota on neurogenesis is most present at a younger age. Supporting data showing a decrease or no change in neurogenesis was provided by Celorrio et al. [37], who studied the importance of microbiota after traumatic brain injury since these patients are susceptible to antibiotic-induced dysbiosis. Neurons in the dentate gyrus of the hippocampus are especially sensitive to neurodegeneration after brain injury. In brain-injured mice in which antibiotics were used to eradicate the gut microbiota, DCX+ and BrdU+ cells were lower than in brain-injured mice with intact microbiota. Thus, the study postulated a neuroinflammatory response triggered by the absence of microbiota to be associated with the hindering of neurogenesis. These data demonstrate that recovery from traumatic brain injury, more specifically neurogenesis, is more difficult in the absence of gut microbiota or its metabolites. Which specific bacteria populations are needed for improved neurogenesis after injury is still to be determined. A second study using antibiotics to eradicate the microbiota in vivo demonstrated how metformin and the microbiota together led to cell proliferation in the central nervous system of mice fed a high-fat diet [38]. In the microbiota-depleted high-fat diet model, BrdU+ cells were decreased compared to the conventional high-fat diet-fed mouse. Furthermore, Ma et al. did a fecal microbiota transplant from high-fat diet metformin mice to high-fat diet germ-free mice. The result showed increased BrdU+ cells in the hippocampus of the transplanted group, indicating that there is an interaction between metformin and microbiota affecting neurogenesis. Independent of the experimental design, both studies [37,38] showed that the depletion of microbiota by antibiotics resulted in reduced neurogenesis.

Twelve results addressed BDNF in the germ-free mouse model (Table 2). Apart from De Palma et al. [43] and Neufeld et al. [30], who reported increased BDNF mRNA levels, all studies reported unchanged or reduced BDNF levels in germ-free mice. Intriguingly, Neufeld et al. [30] measured increased BDNF mRNA in the hippocampus of 8-week-old female mice, a finding that parallels the discovery of Scott et al. concerning neurogenesis. A further similarity to Scott et al. was, as a result of hyperresponsive HPA axis activity, elevated serum corticosterone levels. The results by De Palma et al. [43] were equally interesting as they used maternal separation, a model of early-life stress, to cause depression-like behavior later in life. In the microbiota group, stressed and control mice showed no differences in BDNF mRNA expression in the hippocampus. This may mean that maternal separation stress could not affect BDNF expression, which is quite unlikely since the model is widely used. The experimental design was in keeping with previous studies. No change in BDNF mRNA expression could also mean that the microbiota had a protective effect against the adverse stressors. However, the study discovered, opposite to the assumption of a protective feature of microbiota, stressed germ-free mice express higher BDNF mRNA levels in the hippocampus compared to control germ-free mice. The findings of increased BDNF in both studies are quite controversial, as abnormalities in the HPA axis signaling and exposure to stress are expected to lead to reduced production of BDNF in the hippocampus [44].

Results found by Clarke et al. [45], Diaz Heijtz et al. [46], Gareau et al. [47], and Sudo et al. [48] were in line with BDNF mRNA being reduced in the hippocampus of germ-free mice with hyperresponsive HPA axis activity as they all found low BDNF mRNA. Sudo et al. also discovered early reconstitution of germ-free mice with microbiota at the age of 6 weeks to partially correct the enhanced HPA axis response three weeks later. However, reconstitution at the age of 14 weeks could not reverse HPA response, suggesting there is a postnatal time window during which the microbiota converses with brain development, as proposed by Borre et al. [40]. As previously discussed, Clarke et al. [45] found a sex-specific reduction in BDNF in the hippocampus of germ-free male mice during early life. Unfortunately, the exact age at euthanasia was not given, so a precise comparison to the finding of Scott et al. [36], seeing reduced neurogenesis at four weeks of age in male mice, cannot be made.

Another aspect of BDNF is its involvement in synaptic plasticity. Fröhlich et al. [49] and Zeraati et al. [50] discovered reduced hippocampal BDNF mRNA as well as cognitive impairment in antibiotic-induced germ-free status. Further specified, Zeraati et al. found this antibiotic-induced decrease of BDNF in a model of autoimmune encephalitis, while the controls (antibiotic-treated mice without encephalitis) showed no change [50]. In contrast to these studies, Johnson et al. [51] compared conventional germ-free mice to antibiotic-induced germ-free mice and found that in both models, BDNF levels were not affected.

One study [52] reported unchanged BDNF mRNA levels but reduced BDNF protein levels in the hippocampus of juvenile microbiota-depleted mice. A reason for this finding could be the distribution of BDNF protein away from the translation site through axonal transport [53]. If there is a connection between increased axonal transport and change in microbiota composition is still to be determined.

**Table 2:** Germ-free mice and BDNF.

BDNF	Reference
↓ (protein)	[52]
Unchanged	[54]
↓	[45]
↓	[46]
Unchanged	[51]
↓	[49]
↓ (model of autoimmune encephalitis)	[50]
Unchanged (control to [50])	[50]
↑	[43]
↓	[47]
↑	[30]
↓	[48]

This table shows the significant changes in BDNF levels in germ-free mice. ↓ indicates a decrease, ↑ indicates an increase in BDNF levels; “Unchanged” indicates no change in BDNF. Additional information relevant to the study is given in parentheses.

Overall, studies addressing germ-free status in the rodent model displayed lower BDNF levels and frequently less neurogenesis in the hippocampus. Early-life activation of the HPA axis, differences of sex as well as diet seem to play an important role in microbiota-depleted guts influencing cell proliferation in the hippocampus.

### 3.2. Taxonomic Changes

In twenty-six studies, changes in relative bacterial abundance were found at phylum, family, genus, and species levels. For the changes in bacterial abundance, results are presented as correlations. An increase or decrease in both bacteria and neurogenesis or BDNF expression represents a positive correlation, and an inverse result is a negative correlation. Detailed results regarding the increase or decrease of bacterial abundance can be found in Supplementary Data (Supplementary Tables S1 and S2).

#### 3.2.1. Firmicutes

Most changes in microbial taxa were found on the level of and within the Firmicutes phylum. Six studies [38,55,56,57,58,59] reported a change at the phylum level, which significantly correlated with changes in the immunohistological markers of neurogenesis (Table 3). In agreement with data pinpointing the bacterial effects on neurogenesis, results regarding BDNF levels were affirmative to neurogenesis with more than twice as many negative correlations as positive ones (Table 4).

As already thematized in the germ-free chapter, data regarding a high-fat diet was also found in relation to the Firmicutes phylum. Ribeiro et al. [57] and Ma et al. [38] conducted experiments with mice fed a high-fat diet for 24 weeks. Both studies reported hippocampal neurogenesis to be reduced but found opposing results regarding the abundance of the Firmicutes phylum. High-fat diet-induced obesity has been associated with cognitive impairment and decreased neurogenesis in multiple neurogenic niches of the brain [64,65]. Further research has shown that offspring from obese dams had lower hippocampal DCX density [66]. As for the Firmicutes phylum, an increase in bacterial abundance was related to obesity, partly explainable through the many SCFA-producing species belonging to this phylum which may contribute to increased energy needs and lipogenesis in the liver of obese animals [67]. Although Ribeiro et al. did not see the increase in Firmicutes phylum, they detected higher levels of propionate metabolites in the liver of high-fat diet-fed mice. They further discovered how SCFAs induce depletion of adult neurogenic niches through the mitochondrial by-product, reactive oxygen species (ROS), adding new understanding to the regulation of neurogenesis through the gut microbiota [57].

Major depressive disorder is a key modulator of adult neurogenesis and BDNF levels [10,11]. Three reports [47,59,68] studied the effect of unpredictable chronic mild stress (a model of stress-induced depression) on the gut microbiota and neurogenesis or BDNF. DCX+ cells and Firmicutes abundance were reported to be reduced in one study [59], while the other two studies found reduced BDNF levels parallel to increased Firmicutes abundance [47,68]. Several systematic reviews concluded that there is no consensus in the abundance of Firmicutes phylum in models of depression supporting the irregularities seen in our results [69,70,71].

As reported before, adverse events during early life can result in morbidities later in life. Two studies [58,72] reported the effects of disrupted microbiota through gastrointestinal infection. Hennessey et al. [58] studied infection of neonatal mice with enteropathogenic *Escherichia coli*, discovering reduced Firmicutes phylum to benefit DCX+ cells and BDNF. Furthermore, Jang et al. caused gastrointestinal inflammation and disruption of the microbiota through 2,4,6-trinitrobenzene sulfonic acid (TNBS, known to cause immunogenic reactions) in adolescent mice. They found reduced Firmicutes parallel to reduced BDNF levels. Here, for the first time, the evidence is presented that not only in early life but also during adolescence, the microbiota converses with the brain. Flemer et al. [73] studied the variation of the microbiota in healthy laboratory rats over their lifespan. They discovered that after birth, the Firmicutes phylum is rather low and peaks during adolescence. Of the studies discussing the effects of early life infections, in the study by Jang et al. [72], Firmicutes were low during this critical window, possibly explaining the reduction of BDNF mRNA.

Five studies [55,74,75,76,77] explored how the oral administration of antibiotics at different ages and for different time periods affected neurogenesis and Firmicutes abundance. Keogh et al. [55] and Kayyal et al. [77] investigated how short-term neonatal antibiotic treatment would shape the microbiome and impact the microbiota-gut-brain axis. Compared to controls, the treated mice showed higher Firmicutes abundance parallel to lowered neurogenesis [55] and BDNF [55,77] in both studies. Desbonnet et al. [74] explored the effects of antibiotic treatment in adolescent mice for 7 weeks and found BDNF and Firmicutes to be reduced, contradicting the first two studies. Another study [76] treating adolescent mice for only 2 weeks found the same results as Desbonnet et al. The last study [75] administered antibiotics to adult animals for 13 weeks to investigate the effects of chronic treatment. This study reported a lower abundance of Firmicutes phylum to be beneficial for BDNF levels supporting the negative correlation reported by Keogh et al. [55] and Kayyal et al. [77]. These studies suggest that taxonomic change affects the brain, dependent on age. Previous research has shown that the Firmicutes phylum peaks during the crucial period of adolescence and reduces with advancing age [73,78]. In the studies reviewed, the bacterial abundance of the Firmicutes phylum did not follow the timely pattern of a healthy rodent microbiota. In the postnatal period, a high abundance was found, whilst during adolescence, Firmicutes were reduced. Two studies sequenced the microbiota of elderly mice, but only Kundu et al. [60] found the Firmicutes phylum to be decreased. Unexpectedly, neurogenesis and BDNF mRNA were increased in these elderly mice, although both are known to decline with age, pinpointing a possible technical error as the explanation for this discrepancy. Although the other study [79] saw a decline in BDNF levels, the Firmicutes phylum was increased.

**Table 4:** Firmicutes and BDNF.

Taxa	BDNF			Reference
**Firmicutes**	4	9	2	[47,55,58,60,68,72,74,75,76,77,79,80,81,82,83]
Bacillaceae		1		[55]
Caldicoprobacteraceae			1	[84]
*Caldicoprobacter*			1	[84]
Clostridiaceae		1		[55]
*Candidatus Arthromitus*			2	[82]
*Clostridium*		1	1	[82,85]
Clostridiales vadin BB60	1			[55]
*Coprobacillus*			1	[82]
*Dehalobacterium*			1	[82]
Enterococcaceae		1		[55]
*Enterococcus*		1	1	[86]
Erysipelotrichaceae		2		[55,81]
*Allobaculum*		3	1	[68,79,81,84]
Eubacteriales Family XIII	1			[55]
Lachnospiraceae	2	4	1	[55,58,72,75,76,83,87]
*Anerostipes*		1		[85]
*Blautia*		1	1	[82,85]
*Eubacterium, r = rectale*		1 *(r)*		[47]
*Lachnobacterium*	2			[68,76]
*Lachnospira*	1			[76]
*Lachnospiraceae unspec.*	1	1	2	[43,60,86]
*Roseburia*	1		1	[76,84]
Lactobacillaceae	2			[55,58]
*Lactiplantibacillus, p = plantarum*	1 *(p)*			[88]
*Lactobacillus, b = brevis, h = helveticus, i = intestinalis, j = johnsonii, r = rhamnosus*	10 *(+b, h, I, j, r)*	3 *(+j)*	2	[60,68,72,79,80,81,82,86,88]
Mogibacteriaceae			1	[82]
Paenibacillaceae		1		[55]
*Peptococcocus*			1	[84]
Peptostreptococcaceae			1	[82]
Ruminococcaceae/Oscillospiraceae	2	2	1	[55,58,75,76,83]
*Eubacterium*		1		[76]
*Oscillospira*	1			[76]
*Ruminococcus*			2	[82,83]
*Ruminococcaceae unspec.*	1		1	[86]
*Staphylococcus*			1	[84]
*Streptococcus*			1	[82]

This table shows the positive or negative correlations between the relative changes in the Firmicutes **phylum (bold)**, family (straight), *genus and species (italic)*, and BDNF levels. Green cells indicate a positive correlation, orange cells indicate a negative correlation, and grey cells indicate no change of BDNF with the change of bacterial taxa. Numbers show how many studies were found for each correlation. *(+letter)* with the letter being the first letter of the species name, which symbolizes that a change was found at the genus and species level; *(letter)* means a change was found only at the species level.

Within the order Bacillales, an increase in the abundance of family Bacillaceae [55,61] and family Paenibacillaceae [55] was consistently associated with lower neurogenesis and BDNF levels. Dunphy-Doherty et al. found the same association between the genus *Bacillus* and neurogenesis [61]. Well-studied members of the *Bacillus* genus, *B. anthracis*, *B. cereus*, and *B. thuringiensis*, are known for their pathogenic characteristics, like the synthesis of anthrax and food poisoning agents [89]. In children with celiac disease, a higher abundance of the family, Bacillaceae, was found in patients suffering from abdominal pain and diarrhea [90]. Altogether, these findings underline the association of Bacillaceae and *Bacillus* with poor health and could explain, at least partially, why neurogenesis and BDNF were negatively affected.

Within the order of Eubacteriales, the family Peptostreptococcaceae exhibited a positive correlation with neurogenesis in one study [58]. Family Clostridiaceae was found to negatively correlate with the extent of neurogenesis [55,57] or BDNF levels [55] in all but one study [61]. Additionally, an unspecified genus of the family Defluviitaleaeceae [61] and genus *Eubacterium* [61], both belonging to the order of Eubacteriales as well as genus *Clostridium* [85] and an unspecified genus of family Clostridiaceae [57] supported the negative association in three studies. Although there was an overall negative correlation found at the family and genus level and the family of Clostridiaceae contains pathogenic members like *C. perfringens* or *C. difficile*, it is important to mention that the naturally residing Clostridia plays a beneficial role in maintaining normal gut homeostasis through modulation of metabolic and immune processes [91]. Furthermore, studies have shown that the probiotic *Clostridium butyricum* features neuroprotective properties and can increase hippocampal BDNF expression [92,93]. Since the family of Clostridiaceae is quite large, analysis of taxonomy at higher resolution could have possibly determined whether the microbiota contained harmful species, explaining the negative findings.

As for Enterococcaceae, the family Enterococcaceae [55] or genus *Enterococcus* [86] was associated with low BDNF mRNA levels in the hippocampus in two studies. Congruent to these findings, fecal microbiota transplant from IBD patients with depressive disorder contained a higher abundance of family Enterococcaceae and caused a decrease of hippocampal BDNF in transplanted mice [94]. While at the phylum level, a concise opinion on the abundance of Firmicutes phylum could not be formed, analysis at higher resolution in the case of Enterococcaceae suggests a detrimental effect on BDNF levels in the hippocampus.

Within the order of Erysipelotrichales, some studies reported lower neurogenesis and BDNF levels with an increase in family Erysipelotrichaceae [55,81] or genus *Allobaculum* [68,79,81] and family Turicibacteriaceae or genus *Turicibacter* [57]. In the study by Ribeiro et al. [57], Turicibacteriaceae and *Turicibacter* positively correlated to neurogenesis after a longer course of the high-fat diet. No studies were found explaining why these taxa of order Erysipelotrichales, especially Erysipelotrichaceae and *Allobaculum* had a negative effect on the hippocampus.

The most abundant family in the murine cecum is Lachnospiraceae [95]. At the family level, studies found a negative correlation between Lachnospiraceae and neurogenesis [55,58]. For BDNF, family-level studies did not show conclusive results, with Lachnospiraceae being altered in both directions (Table 4). However, there were several positive correlations at the genus level. Gao et al. [68] and Guida et al. [76] found the diminishment of the genus *Lachnobacterium* to be unfavorable regarding BDNF levels. These authors further found that the decrease of genus *Lachnospira* and genus *Roseburia* led to the same outcome. Only one study found negative correlations with elevated genus *Anaerostipes* and genus *Blautia* parallel to lower levels of BDNF [85]. The family of Lachnospiraceae, especially *Roseburia*, belongs to the group of strong butyrate producers, which, as previously shown, could have a beneficial effect on neurogenesis [96]. Genus *Lachnospira*, which can also produce butyrate [97], has been associated with higher levels of BDNF [98]. Therefore, multiple genera of the family Lachnospiraceae interact positively with the hippocampus, probably through the production of SCFAs. At the same time, there must be other members of the family hindering cell proliferation or expression of BDNF mRNA, resulting in the bidirectional results found at the family level.

There was a consistent positive correlation between neurogenesis as well as BDNF and bacterial abundance for the family Lactobacillaceae, best known as a probiotic family (Table 3 and Table 4). Only one study reported otherwise with an increase in DCX+ cells parallel to less abundance of species *Lactobacillus johnsonii* [60]. As for BDNF, three studies found the genus *Lactobacillus* [68,79] or species *Lactobacillus johnsonii* [60] to lower BDNF production. Multiple studies have researched the benefits of probiotic *Lactobacillus subspecies* for neurogenesis and BDNF in healthy and depressed mouse models [63,99,100]. Some of the discovered pathways of interaction where the induction of nerve growth factor [99], an increase of hippocampal endocannabinoids [63], and modulation of neuroinflammatory pathways [101].

On the family level, results regarding the Ruminococcaceae family were controversial since almost the same number of studies reported positive resp. negative correlations for neurogenesis [55,58,63] and BDNF [55,58,75,76]. At the genus level, though, results showed exclusively positive correlations for the genus *Oscillospira* [61,76], genus *Eubacterium* [76], genus *Ruminococcus* [37,57], and unspecified Ruminococcaceae genus [57,86]. An in vitro study by Park et al. found the probiotic species *Ruminococcus albus* to have neuroprotective properties on oxidatively stressed SH-SY5Y cells (human-derived neuroblastoma cells) by increasing the expression of BDNF. The genus *Oscillospira* has the potential to be used as a probiotic since it is strongly associated with leanness and lower BMI, which consequently is associated with adult neurogenesis. Additionally, *Oscillospira* possesses the capability of producing SCFAs, predominantly butyrate [102]. The studies reporting negative correlations between the Ruminococcaceae family and neurogenesis or BDNF mRNA had different experimental designs, such as neonatal infection [58], chronic exposure to stress [63], and long-term antibiotic treatment [75]. How these methods interact specifically with Ruminococcaceae and result in negative correlations is unclear since, at the genus level, only positive effects could mechanistically be explained so far.

#### 3.2.2. Bacteroidetes

Like the Firmicutes phylum, studies reported both an increase and decrease in the Bacteroidetes phylum (Table 5 and Table 6). Interestingly, studies showing a change of Firmicutes phylum in one direction reported a change of Bacteroidetes towards the opposite direction, indicating a possible compensatory mechanism between the two phyla. Five studies reported a taxonomic change at the phylum level, of which only three correlated the bacterial abundances with changes in neurogenesis (Table 5). As for BDNF, data varied in both directions, with the positive correlation group only being composed of decreased phylum parallel to decreased BDNF levels. However, six out of 10 studies reported a negative correlation at the phylum level (Table 6).

Hence, results regarding neurogenesis tended towards a positive correlation with the Bacteroidetes phylum (Table 5). On the contrary, results regarding BDNF clearly showed a negative correlation (Table 6).

There were three studies studying neurogenesis and change of Bacteroidetes phylum [38,55,57]. Interestingly the two studies experimenting with high-fat diets found opposing results, with Ribeiro et al. [57] stating a decrease of Bacteroidetes phylum to be favorable for neurogenesis, whilst Ma et al. [38] found a decrease at the phylum level to reduce cell proliferation. Bacteroidetes are known to be reduced in obese mice and have been associated with weight loss in humans [103]. In a previous study, obesity led to impaired neurogenesis because of the accumulation of senescent cells in the subventricular zone [64]; how a whole phylum associates with these findings remains unclear. One could argue that in obese animals, Bacteroidetes are reduced in relation to Firmicutes as a compensatory mechanism since increased Firmicutes abundance has been clearly associated with obesity.

Multiple studies found an increase of Bacteroidetes disadvantageous and a decrease in beneficial BDNF levels (Table 6). Only three of a total of nine studies found a positive correlation between the two. Two of the three studies [55,74] had in common that in studying postnatal exposure to antibiotics, both found reduced BDNF parallel to lower bacterial abundance. In the negative correlation group, all three studies exposed adult animals to antibiotics [75,76,88]. After birth, the gut microbiota of healthy laboratory rats compromises to a large part of Bacteroidetes that reduces at adolescence [73]. The loss of Bacteroidetes in the postnatal antibiotic exposure group could be responsible for the measured dip in BDNF mRNA levels. Also, Bacteroidetes correlated negatively with BDNF in the adult group showing that this phylum has an impact on hippocampal homeostasis early in life.

All studies focusing on the phylum of Bacteroidetes reported bacterial taxa belonging to the order of Bacteroidales. Genus *Parabacteroides* [57], genus *Barnesiella* and genus *Odoribacter* [85] were each reported in a single study to have a positive correlation with neurogenesis and BDNF levels. These data are awaiting confirmation by independent research groups. For four out of the five families discussed, we could not find a definite allocation to one correlation group. Unclear results were reported for the family Bacteroidaceae and genus Bacteroides, family Bacteroidales S24-7, family Prevotellaceae, and genus Prevotella and family Rikenellacceae. Differences and discrepancies between the studies are mentioned in the paragraphs following.

The correlation of the Bacteroidaceae family as well as genus *Bacteroides* altered in both directions, with each correlation group having almost as many studies for neurogenesis as for BDNF (Table 5 and Table 6). While Kundu et al. [60] and Li et al. [79], who both studied age-related shifts in microbiota and its effect on cognition, found an increase of an unspecified species of *Bacteroides* genus to promote neurogenesis and to heighten BDNF levels, three other studies [47,87,88] found an inverse correlation between *Bacteroides* genus and BDNF. In general, genus *Bacteroides* contributes to the resistance against the colonization of enteric pathogens such as *Campylobacter ssp*. Or *Salmonella ssp.*, which can produce SCFAs (propionic acid) and cross-feeds other gut residents, which as a result, produce more SCFAs like butyrate [104]. Taken together, even though *Bacteroides* are beneficial for gut homeostasis, the family Bacteroidaceae and genus *Bacteroides* tended to reduce BDNF mRNA in the adult hippocampus.

As for family Bacteroidales S24-7 and genera of this family, Ribeiro et al. found that after 24 weeks of the high-fat diet, bacterial abundance and neurogenesis were low; at 14 weeks of the high-fat diet, bacterial abundance was low, but on the contrary, neurogenesis was elevated. Regarding BDNF, postnatal short-term antibiotics treatment reduced Bacteroidales S24-7 and hindered BDNF production [55], while chronic treatment with antibiotics for 13 weeks reduced Bacteroidales S24-7 as well but led to better BDNF production [75]. Experimental designs for this family were very heterogeneous, and clear explanations for these results could not be identified.

The available studies regarding the family Porphyromonadaceae tended towards a negative correlation with neurogenesis and BDNF levels [57,63,75]. Porphyromonadaceae strongly associates with reduced visceral adipose tissue [105,106,107], and the family is linked to SCFA production (propionate) [108,109]. The route of this apparent discrepancy is unknown since propionate-producing bacteria of the Firmicutes phylum have been associated with obesity and reduced adult neurogenesis. As a consequence, Porphyromonadaceae should have shown a positive correlation to BDNF.

At the family level of Prevotellaceae, there was a tendency towards a negative correlation between the family and BDNF levels (Table 6). There was no data found regarding neurogenesis and the family Prevotellaceae. Genus *Prevotella* and an unspecified genus of the Prevotellaceae family both showed a negative correlation in all but two mentioned studies. Less abundance of genus *Alloprevotella* positively correlated with reduced BDNF. What is known is that genus *Prevotella* and *Alloprevotella* belong to the SCFA-producing (acetate and propionate) bacteria and, therefore, should not, at least at the genus level, lead to impaired neurogenesis or BDNF mRNA in the hippocampus [31].

For and within the family of Rikenellaceae, results were conflicting because studies regarding neurogenesis tended towards a negative correlation, and studies regarding BDNF levels found a positive correlation with bacterial abundance (Table 5 and Table 6). There were two studies [58,72] investigating the impact of intestinal inflammation on the microbiome and alterations in the brain. Interestingly, they found opposing results regarding Rikenellaceae abundance. A major difference between these studies was the age at which experiments were carried out. The study identifying a positive correlation observed the results in adolescent mice, while the negative correlation between Rikenellaceae and neurogenesis was seen in neonatal mice. This finding is conflicting with the higher taxonomic rank, the phylum level since Bacteroidetes are more abundant after birth and subside with age [73]. With three out of four studies, genus *Alistipes* tended towards positive correlation. Previous research regarding the family Rikenellaceae and genus *Alistipes* is just as contrasting as the results found in the studies reviewed. Especially genus *Alistipes* has been found to parallel inflammation and depression in humans but may as well have protective effects against liver fibrosis, colitis, and cardiovascular disease [110].

#### 3.2.3. Proteobacteria

Phylum Proteobacteria was reported to have a negative correlation with neurogenesis and BDNF in multiple studies, but few studies also reported the opposite (Table 7 and Table 8). Furthermore, all studies reporting a negative correlation between phylum and neurogenesis or BDNF found an elevation of Proteobacteria to be damaging [55,57,72,74,76].

Studies experimenting with antibiotic treatment clustered differentially. Other than mentioned before, not age but the time span of antibiotic treatment was the distinctive factor, and independent of the exposure duration, Proteobacteria abundance was increased in all four studies. Hoban et al. [75] treating mice with antibiotics for 13 weeks showed opposite results (positive correlation), especially for BDNF, to the studies giving antibiotics for a short period of time [55,74,76]. A reason for why there was a positive correlation between BDNF and Proteobacteria after chronic treatment with antibiotics was not found. All studies used a combination of antibiotics which are poorly absorbed systemically and, therefore, should not have a direct effect on brain physiology [49]. Previous research found ampicillin exposure to suppress BDNF expression and cause neuroinflammation in the hippocampus by a Proteobacteria-dominant dysbiosis in mice [111]. However, ampicillin has also been proven not to cross the blood–brain barrier indicating the changes in the hippocampus to be directly Proteobacteria-related [49]. These findings show that, in antibiotic-induced dysbiosis, the abundance of Proteobacteria is increased. In addition, they suggest that, dependent on the duration of antibiotic treatment, the changes in bacterial abundances cause changes in the hippocampus. It seems plausible to assume that the longer the antibiotic therapy, the more likely it is that neurogenesis or BDNF mRNA is reduced. However, in the studies reviewed, we found even short-term treatment with antibiotics to lead to a negative correlation with BDNF.

At a higher-resolution taxonomic rank in the order of Burkholderiales, the families and genera exhibited varying associations with neurogenesis and BDNF expression. The order of Burkholderiales contains the four important families of Alcaligenaceae, Burkholderiaceae, Oxalobacteraceae, and Sutterellaceae.

At the family level, Oxalobacteraceae [68] correlated negatively with BDNF levels, while the family Alcaligenaceae [55] correlated positively with neurogenesis and BDNF in single studies.

Genus *Parasutterella* and genus *Cupriavidus*, partly specified as *Cupriavidus metallidurans* and an unspecified species of order Burkholderiales, showed a positive correlation [60] for neurogenesis and BDNF levels. However, Gao et al. [68] stated the opposite at the genus level. Comparisons between these three studies are difficult because of the different study designs. The study designs differed in mice age and experimental methods, with one study evaluating the effects of antibiotic treatment and the other studying the impact of chronic stress on BDNF expression.

Not belonging to the order Burkholderiales is the family Enterobacteriaceae. The family and its higher taxonomic resolution had a consistent negative correlation with neurogenesis and BDNF levels in all reviewed studies. The reduction of the genus *Klebsiella* and genus *Shigella* [88] led to an increase, and the increase of the species *Escherichia coli* [72] led to a decrease in BDNF levels. The members of the Enterobacteriaceae family are well known for causing various syndromes and diseases, such as foodborne diarrhea, enteritis, colitis, hemolytic-uremic syndrome, and extraintestinal diseases [112]. A confirmation of this negative correlation was presented in a study in which increased *Shigella* positively correlated with increased microglia activation (a marker of neuroinflammation) and decreased hippocampal neurogenesis in offspring from obese dams [66].

#### 3.2.4. Actinobacteria

Studies regarding the Actinobacteria phylum reported heterogeneous changes regarding an effect on neurogenesis as in the other three phyla (Table 9). Similar to neurogenesis, five studies reported a decrease or increase at the Actinobacteria phylum level, which correlated with changes in BDNF in the hippocampus (Table 10).

Studies concerning phylum Actinobacteria showed a regularity with a positive correlation in six of ten studies (Table 9 and Table 10). Furthermore, the family Coriobacteriaceae [55], family Eggerthellaceae [58] as well as several genera of this phylum [37,88] demonstrated positive correlation in single studies. Despite representing a minority of commensal bacteria in the gut, Actinobacteria play a vital role in maintaining gut permeability, cross-feeding other butyrate-producing bacteria, down-regulating inflammatory processes, and direct involvement with neural mechanisms [113]. These features of the Actinobacteria phylum are in accordance with the positive results we found.

Also known as a probiotic, most studies reported a positive correlation with neurogenesis and BDNF within the family Bifidobacteriaceae, further specified as genus *Bifidobacterium* [57,72,88] and species *Bifidobacterium longum* [80,86]. Studies have shown that probiotic strains of *Bifidobacterium* with and without a combination of other probiotics could increase BDNF in the hippocampus. Still, the link between probiotics and neurogenesis remains unresolved [114]. Three studies [57,68,81], however, reported a bacterial abundance of family Bifidobacteriaceae and genus *Bifidobacterium* to have negative effects on neurogenesis and BDNF mRNA levels, *inter alia* under high-fat diet [38,57]. This implies that the modulation of neurogenesis and BDNF in the obese mouse model by Bifidobacteriaceae family or even Actinobacteria may play a subordinate role or may even be independent of these taxa since *Bifidobacteria* as a probiotic is generally associated with better health.

## 4. Discussion

The data on the effects of microbiota on neurogenesis and neurotrophic factors highlight that research in this field is still quite limited. All studies were conducted in preclinical rodent models. The search for neurotrophic factors only delivered reports on BDNF since bacterial influences on other factors have scarcely been researched [46,54].

Our data show that reproducibility at a higher taxonomic resolution is low, with changes in bacterial abundance found mostly in single studies. Only in germ-free mice taxonomic changes at higher resolution resulted in more concise conclusions (Table 11). It is comprehensible that Lactobacillaceae and *Bifidobacterium*, two thoroughly researched families containing many probiotic strains, belonged to the taxa with the most positive correlations with neurogenesis and BDNF levels. Regarding Actinobacteria and the genus *Alloprevotella*, the basis of the positive outcome could not be found. Both phylum and genus are known to produce butyrate and generally are associated with good health. Still, a paucity of studies linking these bacteria to cerebral health makes it difficult to reach a conclusive verdict. Firmicutes and Bacteroidetes phyla, of which the intestinal microbiota is generally compromised, correlated strongly negatively towards neurogenesis and BDNF levels. As neurogenesis is not a common feature of the adult brain, this finding may not be surprising.

Further studies at a higher taxonomic resolution would be of importance to get a clearer picture of the bacterial composition harming or supporting adult hippocampal neurogenesis. In addition to the limited number of well-powered studies, the second limitation of our study was the review of very heterogeneous studies with different study designs, interventions, and other confounding factors. Therefore, standardized studies would be instrumental for future research to simplify comparisons.

Although studies utilizing germ-free mice were also subject to various confounding factors, a more definite conclusion could be made. Most reports stated BDNF and neurogenesis be lowered in the hippocampus of germ-free mice.

In general, the availability of germ-free mice and the option to introduce one or a few bacterial species, creating a gnotobiotic model, was instrumental to understanding microbe-microbe but also microbe-host interactions. In combination with further advancements like 16s rRNA and metagenomic sequencing, even microbiota-host interactions of unculturable species can be studied.

Total depletion of the gut microbiota by antimicrobial therapy consistently led to a decrease in neurogenesis and BDNF expression, confirming that the presence of an intact microbiome is an important determinant of normal neurogenesis and BDNF expression in adults. Some studies conducted experiments with altered fecal microbiota transplantation (FMT) to germ-free mice to demonstrate the significance of the gut bacteria. For example, Kundu et al. transplanted the microbiota of old-aged mice to young mice and discovered an improvement in neurogenesis in young mice through this microbiota composition [60]. The interaction between diet, metformin, and the microbiota was demonstrated by Ma et al. [38]. They administered the feces of high-fat diet metformin-treated mice to germ-free mice and found increased hippocampal neurogenesis compared to the FMT without metformin.

As for age, events during early life up to the age of eight weeks, meaning almost until the end of rodent adolescence [115], were consequential for neurogenesis and BDNF level modulation. Parallel to continuing brain development, most of the gut colonization begins at birth, and a complex adult-like microbiota is formed by the age of one. With age, the microbiota changes in composition and especially loses its diversity [116]. That early life is indeed a critical time window was confirmed by experiments reconstituting germ-free mice with microbiota in adulthood, and the discovery that changes in cell proliferation could not be affected after a certain age anymore.

Age effects were partially gender-dependent. Although differences in neurogenesis in germ-free male and female rodents normalized in adulthood, male mice showed an early decline of neural cell proliferation at four weeks, while female mice showed a significant increase at eight weeks of age. The differences in adulthood subsided, and the first weeks of life have already been discussed to be a crucial time for determining adult neurogenesis. Therefore, it could be interesting to divert attention toward the brain physiology between birth and adulthood, such as the process of apoptosis and axon pruning, to study if differences can be found between males and females. In mammals, neurons are produced in excess during embryogenesis. Neurons that are unable to form connections and synapses are redundant and are eliminated through these two processes [117]. Studies so far, however, have not been able to define sex-specific differences in brain development, leaving it uncertain whether there is a sex-pruning interaction and whether the microbiota plays a role in this process.

A characteristic found commonly in germ-free mice is elevated corticosterone levels as an indicator of a hyperresponsive HPA axis. This was found parallel to reduced BDNF mRNA in the hippocampus in multiple studies. Changes in both factors are associated with depression. Still, so far, no study has elucidated the exact mechanisms of interaction between microbiota, HPA axis activation, and neurogenesis or BDNF levels, respectively.

Age was a topic that came up repeatedly during the review of the studies reporting taxonomic change. While the Bacteroidetes phylum seems to be more abundant and important early in life, the significance of the Firmicutes phylum rises during the second modulatory window, adolescence. The importance of these phyla was confirmed in mice showing lower BDNF levels after postnatal depletion of Bacteroidetes and analog results after Firmicutes depletion during adolescence. In addition, Proteobacteria-dominant dysbiosis during infancy led to reduced BDNF, but higher abundance during adulthood exerted no effects. Although age seems to be a main cofounder at the phylum level, it could not be traced to higher taxonomic resolution. Only two studies found an increase within the *Bacteroides* genus to promote neurogenesis in aged mice.

We identified a second important theme in several studies, namely the association between SCFA-producing bacteria, obesity, and obesity’s negative effects on the hippocampus. In recent literature, butyrate administration has been regarded as a possible prevention strategy against obesity [118], while propionate has been associated with liponeogenesis and acetate to cholesterol synthesis [119]. Contrarily, the Firmicutes phylum, which contains most of the strong butyrate-producers [31,120], is more abundant in obese subjects and, in our review, correlated negatively to neurogenesis and BDNF. Whether or not the high abundance of Firmicutes phylum in obese subjects represents a compensatory mechanism is being debated. Then again, the genus *Oscillospira* (belonging to the Firmicutes phylum) is a butyrate-producer, strongly associated with lower BMI, and showed exclusively positive correlations in this review. Pathways of microbial propionate formation have been found predominantly in the Bacteroidetes phylum [121], which conversely is lower in obese subjects and has even been associated with weight loss. Still, we found that the phylum was in negative correlation to BDNF expression in the hippocampus. Data regarding the family Porphyromonadaceae was also puzzling, with the negative correlation being in line with propionate production [109] but Porphyromonadaceae being associated with low visceral adipose tissue [105]. Only genus *Bacteroides* followed a comprehensible pattern with being a propionate-producer [31], is increased in overweight subjects [119] and showing a negative correlation towards BDNF. In summary, the effects of obesity-dependent microbiota and its metabolites on neurogenesis and BDNF differ from phylum level to a higher taxonomic resolution, leaving unsolved which and how much SCFAs are beneficial and whether an allocation to a particular bacterial taxon is possible. Furthermore, there are many more bacterial metabolites playing a role in the microbiota-gut-brain axis [31]. These could also converse with the functions of SCFAs, causing a gain or loss of function and confounding the actual effects on neurogenesis and BDNF expression.

In several studies, antibiotics were used to deplete or cause a perturbation in the microbiota. Mostly, the antibiotics chosen were poorly absorbed in the gastrointestinal system to minimize interactions with host physiology. At the phylum level of Proteobacteria, we discovered that in three studies, the short-term antibiotic treatment caused a Proteobacteria-dominant dysbiotic gut and led to a decrease of BNDF in the hippocampus, signifying there is an interaction between antibiotics, Proteobacteria, and processes in the hippocampus.

In conclusion, strong negative correlations towards BDNF expression were found with germ-free mice and at the phylum level of Firmicutes, Bacteroidetes, and Proteobacteria. For phylum Firmicutes, there was a negative correlation with neurogenesis found as well. While it makes sense that the depletion of healthy gut microbiota, such as in germ-free mice, leaves a damaging impact on the brain, it is a matter of debate why both major phyla of the healthy microbiota (Firmicutes and Bacteroidetes) demonstrated a strong negative correlation. Thus, the outcome at the phylum level should be treated with caution. Further, multiple topics, including age, obesity, chronic stress, and antibiotic treatment, concerning the involvement of the microbiota in neurogenesis and BDNF expression were found. However, a definite connection between a bacterial taxon and BDNF expression or neurogenesis in adult animals could not always be made, resulting in, at times, inconclusive results. Here, studies with gnotobiotic mice focusing their interest on one taxonomic rank of bacteria would be helpful to determine the individual interactions with the microbiota-gut-brain axis. The results of the single-strain studies would not be directly projectable onto the microbiota since bacterial cultures communicate among themselves. Thus, for a more realistic picture, the microbiota must be looked at as a whole. As the number of studies on the microbiota-gut-brain axis continues to grow, the research on the interaction between the microbiota and neurogenesis will lead to a more detailed understanding of bacterial roles in adult neurogenesis. Especially the research on the relation of neurotrophic factors other than BDNF to microbiota could be of interest to future studies. Human interventional trials investigating the effects of probiotics on depression and serum BDNF are already arising [122,123,124]. It is tempting to assume that further such studies may help to work towards better healing of damaged neurons as a result of aging or disease.

## Figures and Tables

**Table 1:** Germ-free mice and neurogenesis.

Neurogenesis	Reference
↓ (DCX/BrdU; male, 4 weeks)	[36]
Unchanged (male, 8 weeks)	[36]
Unchanged (male, 12 weeks)	[36]
Unchanged (female, 4 weeks)	[36]
↑ (DCX/BrdU; female, 8 weeks)	[36]
Unchanged (female, 12 weeks)	[36]
↓ (DCX/BrdU)	[37]
↓ (BrdU)	[38]
↑ (BrdU)	[39]

This table shows the significant changes in DCX and BrdU immunohistology of neurogenesis in germ-free mice. ↓ indicates a decrease, ↑ indicates an increase in neurogenesis; “Unchanged” indicates no change in neurogenesis. Additional information relevant to the study is given in parentheses.

**Table 3:** Firmicutes and neurogenesis.

Taxa	Neurogenesis			Reference
**Firmicutes**	2	4	1	[38,55,56,57,58,59,60]
Bacillaceae		2		[55,61]
*Bacillus*		1		[61]
Clostridiaceae	1	2		[55,57,61]
*Butyricicoccus*			1	[56]
*Clostridiaceae unspec.*		1		[57]
Clostridiales vadin BB60	1			[55]
Defluviitaleaeceae unspec.		1		[61]
Enterococcaceae		1		[55]
Erysipelotrichaceae		1		[55]
Eubacteriales Family XIII	1	1		[55,58]
*Eubacterium*		1		[61]
Lachnospiraceae		2	1	[55,56,58]
*Lachnospira*			1	[62]
*Lachnospiraceae unspec.*	1	1		[60,61]
*Marvinbryantia*		1		[55]
Lactobacillaceae	3			[55,58,63]
*Lactobacillus, i = intestinalis, j = johnsonii*	1 *(+i)*	1 *(j)*		[38,60]
Paenibacillaceae		1		[55]
Peptostreptococcaceae	1			[58]
Ruminococcaceae/Oscillospiraceae	1	2	1	[55,58,62,63]
*Oscillospira*	1		1	[61,62]
*Ruminococcus*	2	1		[37,57]
*Ruminococcaceae unspec.*	1		1	[56,57]
Turicibacteraceae	1	1		[57]
*Turicibacter*	1	1	1	[56,57]
Veillonellaceae		1		[61]
*Veillonella*		1		[61]

This table shows the positive or negative correlations between the relative changes in the Firmicutes **phylum (bold)**, family (straight), *genus and species (italic)*, and neurogenesis. Green cells indicate a positive correlation, orange cells indicate a negative correlation, and grey cells indicate no change of neurogenesis with the change of bacterial taxa. Numbers show how many studies were found for each correlation. *(+letter)* with the letter being the first letter of the species name, which symbolizes that a change was found at the genus and species level; *(letter)* means a change was found only at the species level.

**Table 5:** Bacteroidetes and neurogenesis.

Taxa	Neurogenesis			Reference
**Bacteroidetes**	2	1	2	[38,56,57,60,62]
Bacteroidaceae	1	1		[55,57]
*Bacteroides, u = unspec.*	1 *(u)*			[60]
Bacteroidales S24-7	2	1		[55,57]
*Bacteroidales S24-7 unpec.*	1	1		[55,57]
*Parabacteroides*	1			[57]
Poryphyromonadaceae	1	1		[57,63]
Prevotellaceae			1	[62]
*Alloprevotella*			1	[56]
*Prevotella*		1		[60]
*Prevotellaceae unspec*		1		[61]
Rikenellaceae	1	1		[55,58]
*Alistipes*		2		[57,60]

This table shows the positive or negative correlations between the relative changes in the Bacteroidetes **phylum (bold)**, family (straight), *genus and species (italic)*, and neurogenesis. Green cells indicate a positive correlation, orange cells indicate a negative correlation, and grey cells indicate no change of neurogenesis with the change of bacterial taxa. Numbers show how many studies were found for each correlation. *(letter)* with the letter being the first letter of the species name, symbolizing that a change was found at the species level only.

**Table 6:** Bacteroidetes and BDNF.

Taxa	BDNF			Reference
**Bacteroidetes**	3	6	1	[55,68,72,74,75,76,79,80,83,88]
Bacteroidaceae	1	2		[55,75,87]
*Bacteroides, u = unspec.*	1 *(+u)*	3		[47,60,79,87,88]
Bacteroidales S24-7	1	1	3	[55,75,82,83]
*Bacteroidales S24-7 unpec.*			1	[83]
*Barnesiella*	1			[85]
*Odoribacter*	1			[85]
Poryphyromonadaceae		1	1	[75,84]
Prevotellaceae	1	2		[68,74,75]
*Alloprevotella*	2		1	[85,86]
*Prevotella*	2	2	2	[60,68,79,82,85]
Rikenellaceae	3	1	2	[55,72,74,75,82]
*Alistipes*	1	1		[60,85]

This table shows the positive or negative correlations between the relative changes in the Bacteroidetes **phylum (bold)**, family (straight), *genus and species (italic)*, and BDNF levels. Green cells indicate a positive correlation, orange cells indicate a negative correlation, and grey cells indicate no change of BDNF with the change of bacterial taxa. Numbers show how many studies were found for each correlation. *(+letter)* with the letter being the first letter of the species name, symbolizing that a change was found at the genus and species level.

**Table 7:** Proteobacteria and neurogenesis.

Taxa	Neurogenesis			Reference
**Proteobacteria**	2	2		[55,57,59]
Alcaligenaceae	1			[55]
*Burkholderiales, u = unspec.*	1 *(u)*			[60]
*Cupriavidus, m = metallidurans*	1 *(m)*			[60]
Enterobacteriaceae		1		[55]
*Parasutterella*		1		[60]

This table shows the positive or negative correlations between the relative changes in the Proteobacteria **phylum (bold)**, family (straight), *genus and species (italic)*, and neurogenesis. Green cells indicate a positive correlation, and orange cells indicate a negative correlation with the change of bacterial taxa. The Grey column means no studies were found. Numbers show how many studies were found for each correlation. *(letter)* with the letter being the first letter of the species name, symbolizing that a change was found at the species level only.

**Table 8:** Proteobacteria and BDNF.

Taxa	BDNF			Reference
**Proteobacteria**	2	4		[55,72,74,75,76,80]
Alcaligenaceae	1		1	[55,84]
*Burkholderiales, u = unspec.*	1			[60]
*Cupriavidus, m = metallidurans*	1 *(m)*	1		[60]
Enterobacteriaceae		4	1	[47,55,72,76,82]
*Escherichia, c = coli*		1 *(c)*		[72]
*Klebsiella*		1		[88]
*Shigella*		1		[88]
*Ochrobactrum*		1		[68]
Oxalobacteraceae		1		[68]
*Parasutterella*	1			[60]

This table shows the positive or negative correlations between the relative changes in the Proteobacteria **phylum**, family, *genus, species*, and BDNF levels. Green cells indicate a positive correlation, orange cells indicate a negative correlation, and grey cells indicate no change of BDNF with the change of bacterial taxa. Numbers show how many studies were found for each correlation. *(letter)* with the letter being the first letter of the species name, symbolizing that a change was found at the species level only.

**Table 9:** Actinobacteria and neurogenesis.

Taxa	Neurogenesis			Reference
**Actinobacteria**	3	2		[38,55,58,59,61]
*Atopobium*	1			[37]
Bifidobacteriaceae	2	1		[57,58]
*Bifidobacterium*	1	1		[57]
Coriobacteriaceae	1			[55]
Eggerthellaceae	1			[58]
Nocardiaceae		1		[61]
*Rhodococcus*		1		[61]

This table shows the positive or negative correlations between the relative changes in the Actinobacteria **phylum (bold)**, family (straight), *genus and species (italic)*, and neurogenesis. Green cells indicate a positive correlation, and orange cells indicate a negative correlation with the change of bacterial taxa. The grey column means no studies were found. Numbers show how many studies were found for each correlation.

**Table 10:** Actinobacteria and BDNF.

Taxa	BDNF			Reference
**Actinobacteria**	3	2	1	[55,58,79,81,84,88]
*Actinomyces*	1			[88]
Bifidobacteriaceae	1	2	1	[58,68,81,84]
*Bifidobacterium, l = longum*	4 *(+l)*	2	2 *(+l)*	[68,72,80,81,84,86,88]
Coriobacteriaceae	1		3	[55,82,84]
*Corynebacterium*	1			[88]
*Adlercreutzia*			2	[82]
*Mycobacterium*	1			[88]
*Proprionibacterium*			1	[84]

This table shows the positive or negative correlations between the relative changes in the Actinobacteria **phylum (bold)**, family (straight), *genus and species (italic)*, and BDNF levels. Green cells indicate a positive correlation, orange cells indicate a negative correlation, and grey cells indicate no change of BDNF with the change of bacterial taxa. Numbers show how many studies were found for each correlation. *(+letter)* with the letter being the first letter of the species name, symbolizing that a change was found at the genus and species level.

**Table 11:** Bacterial taxa, with most studies found for positive or negative correlation to neurogenesis and BDNF.

Positive Correlation	Negative Correlation
Lactobacillaceae	**Firmicutes**
*Lactobacillus* (BDNF)	Bacillaceae (neurogenesis)
*Alloprevotella* (BDNF)	Lachnospiraceae
**Actinobacteria**	**Bacteroidetes** (BDNF)
*Bifidobacterium* (BDNF)	Enterobacteriaceae (BDNF)

In this table, we show the bacterial taxa for which we found the most studies for positive and negative correlations. Colored cells indicate that a correlation was found between neurogenesis and BDNF. In the uncolored cells, correlation to neurogenesis or BDNF expression is specified in parentheses. The correlations were found at **phylum (bold)**, family (straight), and *genus (italic)* levels.

## Data Availability

Not applicable.

## Supplementary Materials

The following supporting information can be downloaded at: https://www.mdpi.com/article/10.3390/ijms232415934/s1.

## Abbreviations

DCX	Doublecortin
BrdU	Bromodeoxyuridine
BDNF	Brain-derived neurotrophic factor
SCFA	Short-chain fatty acid
NeuN	Neuronal Nuclei
HPA axis	Hypothalamic-pituitary-adrenal axis
GABA	Gamma-aminobutyric acid
NMDA	N-methyl-D-aspartic acid
ROS	Reactive oxygen species
TNBS	2,4,6-trinitrobenzene sulfonic acid
NGF	Nerve growth factor
NTF	Neurotrophic factor
PSD-95	Postsynaptic density protein 95
FMT	Fecal microbiota transplantation
