# Supplementary material for: Involvement of Intestinal Microbiota in Adult Neurogenesis and the Expression of Brain-Derived Neurotrophic Factor
Source: Int J Mol Sci. 2022 Dec 14;23(24):15934. doi: 10.3390/ijms232415934 (PMC9783874; doi:10.3390/ijms232415934)
Supplement: Supplementary file 1 [file ijms-23-15934-s001.zip › ijms-2066405-supplementary.pdf]

Supplementary

Supplementary Table S1. Changes in bacterial abundance and neurogenesis.

Taxa	[60]	[55]	[56]	[57]	[57]	[58]	[59]	[37]	[62]	[38]	[63]	[61]
**Firmicutes**	↓	↑	↓		↓	↓	↓			↑		
Bacillaceae		↑										↑
Bacillus												↑
Clostridiaceae		↑		↓								↓
Butyricicoccus			↓									
Clostridiaceae unspec.				↓								
Clostridiales vadin BB60		↓										
Defluviitaleaceae un-spec.												↑
Enterococcaceae		↑										
Erysipelotrichaceae		↑	↑									
Eubacteriales Family XIII		x				↓						
Eubacterium						↓						↑
Lachnospiraceae		↑	↓									
Lachnospira									x			
Lachnospiraceae unspec.	↓											↓
Marvinbryantia												↑
Lactobacillaceae		↓				↑					↓	
Lactobacillus, i = intesti-nalis, j = johnsonii	↓*(j)*									↓*(+i)*		

Supplementary Table S1. Cont.

**Taxa**	[60]	[55]	[56]	[57]	[57]	[58]	[59]	[37]	[62]	[38]	[63]	[61]
Paenibacillaceae		↑										
Peptostreptococcaceae						↑						
Ruminococcaceae/Oscil-lospiraceae		↓	↓			↓			x		↑	
Oscillospira									x			↓
Ruminococcus				↓	↓			↑				
Ruminococcaceae unspec.				↑								
Turicibacteraceae				↓	↓							
Turicibacter			↑	↓	↓							
Veillonellaceae												↑
Veillonella												↑
**Bacteroidetes**		↓	↑	↓					↑	↓		
Bacteroidaceae		x			↑							
Bacteroides, u = unspec.	↑*(u)*											
Bacteroidales S24-7		↓		↓	↓							
Bacteroidales S24-7 unpec.				↓	↓							
Parabacteroides				↑								
Poryphyromonadaceae				↑							↑	
Prevotellaceae									↑			
Alloprevotella			↑									

Supplementary Table S1. Cont.

**Taxa**	[60]	[55]	[56]	[57]	[57]	[58]	[59]	[37]	[62]	[38]	[63]	[61]
Prevotella	↓											
Prevotellaceae unspec												↑
Rikenellaceae		x				↓						
Alistipes	↓				↑							
**Proteobacteria**		↑		↑	↑		↓					
Alcaligenaceae		x										
Burkholderiales, u = unspec.	↑*(u)*											
Cupriavidus, m = metallidurans	↑*(m)*											
Enterobacteriaceae		↑										
Parasutterella	↑											
**Actinobacteria**		↓				↑	↓			↑		↑
Atopobium								↑				
Bifidobacteriaceae				↓	↓	↑						
Bifidobacterium				↓	↓							
Coriobacteriaceae		x										
Eggerthellaceae						↑						
Nocardiaceae												↑
Rhodococcus												↑

This table shows detailed change in bacterial abundance of **phylum (bold)**, family (straight), *genus and species (italic)* and neurogenesis. The references are shown in the top row. ↑ indicates increase of bacteria, ↓ decrease of bacteria, x indicates loss of bacteria. Green cells show more neurogenesis, red cells show less and grey cells indicate no change of neurogenesis with change of bacterial taxa. *(+letter)* symbolizes that a change was found at genus and species level; *(letter)* means change was found only at species level.

Supplementary Table S**2.** Changes in bacterial abundance and BDNF levels.

**Taxa**	[60]	[58]	[74]	[55]	[82]	[82]	[75]	[88]	[68]	[72]	[85]	[80]	[86]	[86]	[84]	[83]	[43]	[47]	[76]	[77]	[87]	[81]	[79]
**Firmicutes**	↓	↓	↓	↑	↑		↓		↑	↓		↓				↑		↑	↓	↑		↑	↑
Bacillaceae				↑																			
Caldicoprobacter-aceae															↓								
Caldicoprobacter															↓								
Clostridiaceae				↑																			
Candidatus Athro-mitus					↑	↓																	
Clostridium					↑						↑												
Clostridiales va-din BB60				↓																			
Coprobacillus						↑																	
Dehalobacterium					↑																		
Enterococcaceae				↑																			
Enterococcus													↑	↓									
Erysipelotricha-ceae				↑																		↑	
Allobaculum									↑						↓							↑	↑
Eubacteriales Family XIII				x																			
Lachnospiraceae		↓		↑			↓			↓						↑			↓		↑		
Anarostipes											↑												
Blautia					↑						↑												
Eubacterium rectale																		↑					
Lachnobacterium									x										↓				

Supplementary Table S2. Cont.

Taxa	[60]	[58]	[74]	[55]	[82]	[82]	[75]	[88]	[68]	[72]	[85]	[80]	[86]	[86]	[84]	[83]	[43]	[47]	[76]	[77]	[87]	[81]	[79]
Lachnospira																			↓				
Lachnospiraceae unspec.	↓												↓	↑			↑						
Roseburia															↓				↓				
Lactobacillaceae		↑		↓																			
Lactiplantibacillus plantarum								↑															
Lactobacillus, b = brevis, h = helveticus, i = intestinalis, j = johnsonii, r = rhamnosus	↓ *(j)*					↑		↑ (+ *I, j)*		↓ (+ *j)*		↓ *(+ b, h, r)*	↓	↑								↓	↑
Mogibacteraceae					↑																		
Paenibacillaceae				↑																			
Peptococcus															↑								
Peptostreptococ-caceae					↑																		
Ruminococca-ceae/Oscillospi-raceae		↓		↓			↓									↑			↓				
Eubacterium																			↓				
Oscillospira																			↓				
Ruminococcus					↑											↑							
Ruminococcaceae unspec.													↓	↑									

Supplementary Table S2. Cont.

**Taxa**	[60]	[58]	[74]	[55]	[82]	[82]	[75]	[88]	[68]	[72]	[85]	[80]	[86]	[86]	[84]	[83]	[43]	[47]	[76]	[77]	[87]	[81]	[79]
Staphylococcus															↑								
Streptococcus					↑																		
**Bacteroidetes**			↓	↓			↓	↓	↑	↑		↑				↓			↑				↓
Bacteroidaceae				x			↓														↑		
Bacteroides, u = unspec.	↑ (*u*)							↓										↑			↑		↓
Bacteroidales S24-7				↓	↓	↓	↓									↓							
Bacteroidales S24-7 unpec.																↓							
Barnesiella											↓												
Odoribacter											↓												
Poryphyromona-daceae							↓								↓								
Prevotellaceae			↓				↓		↑														
Alloprevotella											↓		↓	↑									
Prevotella	↓								↑		↓												↓
Rikenellaceae			↓	x	↓	↓	↓			↓													
Alistipes	↓				↓	↓					↓												
**Proteobacteria**			↑	↑			↑			↑		↓							↑				
Alcaligenaceae				x											↓								
Burkholderiales, u = unspec.	↑																						
Cupriavidus, m = metallidurans	↑ (*m*)								↑														

Supplementary Table S2. Cont.

Taxa	[60]	[58]	[74]	[55]	[82]	[82]	[75]	[88]	[68]	[72]	[85]	[80]	[86]	[86]	[84]	[83]	[43]	[47]	[76]	[77]	[87]	[81]	[79]
Enterobacteri-aceae				↑	↑					↑								↑	↑				
Escherichia, c= coli										↑													
Klebsiella								↓															
Shigella								↓															
Ochrobactrum									↑														
Oxalobacteraceae									↑														
Parasutterella	↑																						
**Actinobacteria**		↑		↓				↑							↓							↑	↑
Actinomyces								↑															
Bifidobacteriaceae		↑							↑						↓							↑	
Bifidobacterium, l = longum								↑	↑	↓		↓ (*l*)	↓ (*l*)	↑ (*l*)	↓							↑	
Coriobacteriaceae				x	↑	↑									↓								
Corynebacterium								↑															
Adlercreutzia					↑	↓																	
Mycobacterium								↑															
Propionibacterium															↑								

This table shows detailed change in bacterial abundance of **phylum (bold)**, family (straight), *genus and species (italic)* and BDNF. The references are shown in the top row. ↑ indicates increase of bacteria, ↓ decrease of bacteria, x indicates loss of bacteria. Green cells show higher BDNF levels, red cells show lower and grey cells indicate no change of BDNF levels with change of bacterial taxa *(+letter)* symbolizes that a change was found at genus and species level; *(letter)* means change was found only at species level.
